# Effects of combined traditional processing methods on the nutritional quality of beans

**DOI:** 10.1002/fsn3.209

**Published:** 2015-02-14

**Authors:** Aisha M Nakitto, John H Muyonga, Dorothy Nakimbugwe

**Affiliations:** Department of Food Technology and Nutrition, Makerere UniversityKampala, Uganda

**Keywords:** Antinutrients, dry beans, flour, in vitro protein digestibility, iron and zinc extractability

## Abstract

Consumption of dry beans is limited by long cooking times thus high fuel requirement. The bioavailability of nutrients in beans is also limited due to presence of antinutrients such as phytates and tannins. Little research has been done on combined processing methods for production of nutritious fast cooking bean flour and the effect of combined treatments on nutritional quality of beans has not previously determined. The aim of this study was to reduce cooking time and enhance the nutritional value of dry beans. Specifically to: develop protocols for production of fast cooking bean flours and assess the effect of processing on the nutritional characteristics of the flours. Dry beans (K131 variety) were soaked for 12 h; sprouted for 48 h; dehulled and steamed for 25 and 15 min for whole and dehulled beans respectively or roasted at 170°C for 45 and 15 min for whole and dehulled beans respectively. Dehulling eliminated phytates and tannins and increased protein digestibility. In vitro protein digestibility and mineral (iron and zinc) extractability were negatively correlated with tannin and phytate content. Total available carbohydrates were highest in moist heat-treated bean flours. Overall, combined processing of beans improved the nutritional quality of dry beans and the resulting precooked flours need less cooking time compared to whole dry beans.

## Introduction

Common bean (*Phaseolus vulgaris* L.) is the most important food legume for direct consumption in the world (Jones 2008). East Africa has the highest bean production in sub-Saharan Africa with Tanzania, Kenya and Uganda ranking 7th, 8^th^, and 9^th^, respectively (FAOSTAT 2013). Uganda's annual bean production is about 461,000 MT/Year (FAOSTAT 2013) grown throughout and mainly in the cool southwestern highlands (Opio and Male-Kayiwa 1994). Dry bean consumption is, however, limited by long cooking time and low digestibility. High yielding varieties of beans have been developed in Uganda such as NABE1, NABE2, NABE3, K131, and K132, which could contribute to improve food security but some, such as K131 are prone to Hard-to-Cook (HTC) defect, which leads to high fuel requirement and reduced acceptability and consumption. Development of quick cooking bean flours with increased digestibility and nutrient bio-availability should lead to increased bean consumption thus increased nutritional benefits.

The aim of the study was to develop fast cooking bean flour with enhanced nutritional value. Specifically to: develop protocols for production of fast cooking bean flours and assess the effect of combined processing on the nutritional characteristics of the flours.

## Materials and Methods

### Development of a protocol for processing beans into quick cooking flour

#### Bean sample preparation

K131 (carioca) dry beans, an improved small-seeded variety developed by the Uganda National Crops Research Resources Institute (NaCRRI) were used in this study. While K131 yields up to 3 Mt/ha (UEPB 2005) the beans have a very hard seed resulting in long cooking time (Nyakuni 2008).

K131 beans were obtained from NaCRRI, cleaned, sorted, washed, and disinfected in (0.1%) sodium hypochlorite solution and rinsed in distilled water.

#### Soaking

Five batches of cleaned beans (1000 g) were soaked in distilled water (1:10 w/v) (Ramakrishna et al. 2006; Osman 2007) at room temperature for 6, 12, 24, 48, and 96 h. The weights of the beans were taken after each of the five time durations, and their percentage weight gain calculated. The beans with the highest percentage weight gain were then sprouted.

#### Sprouting

The soaked beans were drained manually, rinsed in distilled water, and divided into three lots that were then sprouted for 24, 48, or 72 h. To sprout, the beans were spread on plastic trays lined and covered with wet cotton cloths, onto which distilled water was sprinkled every 24 h. The beans were examined after each of the sprouting times to determine the time at which radicals appeared long enough but before developing distinct root hairs. The percentage number of beans that sprouted was computed and the sprouting replicated.

#### Dehulling

Half of the sprouted beans were dehulled (seed coats removed manually by hand at room temperature) while the other half was left whole. Half of the dehulled and whole-sprouted beans were separately subjected to either steaming under pressure or roasting.

#### Roasting

Half of the sprouted beans (both dehulled and whole) were roasted in an oven (Infrared food oven GL-2A, Guangzhou Itop Kitchen Equipment Co, Ltd. Guangdong, China (Mainland)) at 170°C for 10, 15, 20, and 25 min. Preferred roasting time was when the beans turned golden brown without burning. The roasted bean seeds were milled into flour (Wonder Mill, Pocatello, ID) and analyzed.

#### Steaming

The second half of the sprouted beans (both dehulled and whole) were steamed over boiling water in a pressure cooker (Sunny; Gujarat, India) for 10, 15, 20, 25, and 30 min. Steaming time was recorded starting after the pressure cooker had attained maximum pressure, that is released steam. The most appropriate steaming time was that at which the beans became soft enough to be crushed by pressing between fingers (Rehman et al. 2001). The steamed beans were then dried at 60°C for 6 h (Infrared food oven GL-2A) and milled into flour (Wonder Mill) for subsequent analysis.

#### Boiling

Boiling was included among the processing methods because it is commonly used to cook beans for home consumption. Beans were sorted, rinsed, placed into a saucepan with distilled water (1:2 w/v), and boiled for 69 min (Nyakuni 2008).

### Chemical analysis

#### Moisture content

Moisture of the whole unprocessed beans as well as that of all the processed flours was determined according to the AOAC (1984) method # 24.003. The results were used to compute iron and zinc extractability, in vitro protein digestibility, total available carbohydrates as well as phytate and tannin levels on dry matter basis.

#### In vitro protein digestibility

Initial protein content was determined using the AACC (1983) Kjeldahl method # 46-12. Approximately 0.5 g of ground sample was weighed into micro-Kjeldahl flasks into which 1 g of Potassium Sulfate, 1 mL of 10% Copper Sulfate, 10 mL of concentrated Sulfuric acid, and boiling beads were added. A blank with no sample was similarly prepared. The flasks were heated on a digestion rack in a fume hood for boiling and for an additional 2 h.

After cooling, the digest was transferred quantitatively to a 50 mL volumetric flask, made to volume with distilled water and mixed immediately. The sample was distilled by pipetting 30 mL into the distillation chamber and slowly adding approximately 30 mL of 50% NaOH solution. A conical flask containing 10 mL of 2% Boric acid solution plus two drops of bromocresol green-methyl red indicator was placed under the condenser stem to collect the distillate. Titration was done using a burette filled with 0.05 N H_2_SO_4_ solution. The percentage crude protein (% CP) in the sample was calculated. 

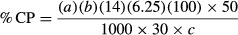


where;

*a* = normality of the acid, that is, approximately 0.05;

*b* = volume of standard acid used (ml), corrected for the blank (i.e., the sample titer minus the blank titer);

*c* = sample weight (mg);

6.25 = conversion factor for protein from % nitrogen; 14 is the protein conversion factor; 50 is the dilution factor; 30 is the volume of distillate and titrate used.

In vitro protein digestibility was then obtained by pepsin digestion according to the method by Mertz et al. (1984). 

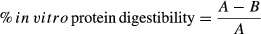


where; *A* = % Protein in sample before digestion, *B* = % Protein in sample after pepsin digestion.

#### Total available carbohydrate

The total available carbohydrate was analyzed by the method of Clegg (1956) and Rehman (2007). To 10 mL of the sample extract 100 mL water was added from which 1 mL of the diluted filtrate was pipetted into a test tube. Duplicate blanks consisting of 1 mL of water each and duplicate standards consisting of 1 mL of dilute glucose each were pipette into test tubes. To each tube, 5 mL of freshly prepared anthrone reagent was rapidly added. All the tubes were stoppered and the contents mixed thoroughly. The tubes were placed in a boiling bath for 12 min and then cooled quickly to room temperature. The solutions were transferred to 1-cm glass cuvettes and the absorbance of the samples and standards read at 630 nm against the reagent blanks (the green color stable at least for 2 h). 

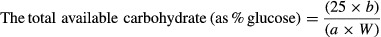


where “*W*” is weight (g) of sample; “*a*” is absorbance of dilute standard; and “*b*” is absorbance of sample.

#### Iron and Zinc extractability

Determination of total and extractable Zinc and Iron content was done based on the method of Duhan et al. (2004). To determine total Zinc and Iron content, one gram of flour sample was placed in a 150 mL conical flask. To this, 30 mL diacid mixture (HNO_3_:HClO_4_; 5:1 v/v) was added and kept overnight. The next day the sample was digested by heating until clear white precipitates settled at the bottom. The crystals were dissolved by diluting in double distilled water. The contents were filtered through Whatman # 42 filter paper. The filtrate was made to 50 mL with double distilled water and used for determination of total Zn and Fe with an atomic absorption spectrophotometer (Perkin-Elmer 3100, Artisan Technology Group 101E Mercury Drive. Champaign, IL 61822, Illinois, USA).

HCl-extractable zinc and iron was determined by the method of Duhan et al. (2004). To 1 g sample, 50 mL 0.03 N HCl was added. The mixture was incubated at 37°C in a shaking water bath (Grant OLS200, Cambridge, UK) for 3 h to simulate conditions that occur in the human stomach. The mixture was then filtered through an ash-less filter paper (Whatman # 42). The filtrate was oven-dried and digested in the diacid mixture. This was followed by the determination of zinc and iron with an Atomic Absorption Spectrophotometer (Perkin-Elmer). Percentage extractability was calculated as follows; 

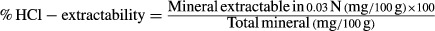


#### Antinutrients

Tannin content was determined according to the vanillin-HCl method (Price et al. 1978) while phytate content was determined using the AOAC (1995) anion-exchange method, number 986.11.

### Statistical analysis

The data were analyzed using SPSS (Statistical Package for Social Scientists) (SPSS Inc. Released 2008. SPSS Statistics for Windows, Version 17.0. Chicago: SPSS Inc.) Version 17. Means and standard deviations were computed. Experimental and sensory evaluation data were analyzed using analysis of variance (ANOVA) at 5% probability level. Duncan's multiple test was used to separate means. Correlation analysis was done to determine the relationship between antinutrients levels and protein digestibility, using Pearson's coefficient.

## Results

### Protocol for production of quick cooking bean flours

#### Soaking

**Figure 1:**
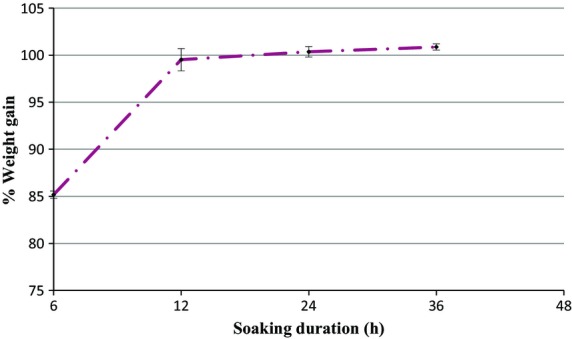
Percentage weight gain of beans over a 48 h soaking period.

The weight of the bean samples increased during soaking (Fig.1) and as the soaking time increased from 6 to 12 h after which the weight only increased slightly.

The beans selected for subsequent processing (sprouting) were those soaked for 12 h.

#### Sprouting

**Figure 2:**
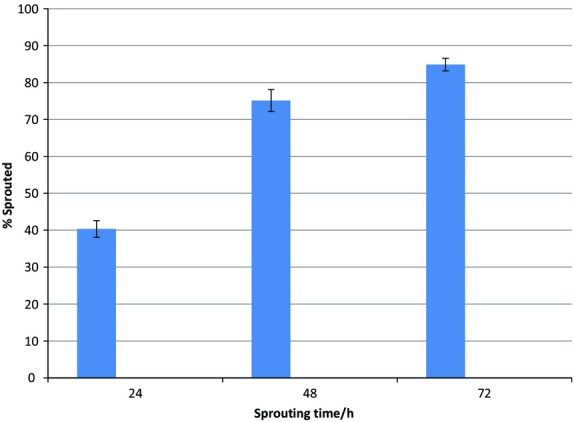
Percentage of beans that sprouted after 24, 48, and 72 h following 12 h of soaking.

The highest percentage of sprouting was recorded after 72 h (Fig.2) after which no more beans sprouted, whereas those that had sprouted germinated. The percentage of beans that had sprouted after 48 h was not significantly different from those that had sprouted after 72 h. Sprouting for 48 h was therefore selected as preferred time.

#### Steaming

For dehulled and whole beans, 15 and 25 min were the most appropriate steaming times, respectively, to be fully cooked.

#### Roasting

The appropriate roasting time for dehulled and whole beans was 15 and 45 min, respectively. After this time, approximately 90% of the beans had attained a golden brown color without being burnt.

### Effect of combined processing methods on the nutritional quality of bean flours

#### Tannin content

Soaking and sprouting followed by dehulling effectively reduced tannins and phytates to below detection (Table1). On the other hand, beans that were similarly processed but not dehulled had high tannin and phytate levels. Similar results were previously reported (Alonso et al. 2000; Egounlety and Aworh 2003; Ghavidel and Prakash 2007).

**Table 1:** Effect of combined processing methods on tannin content of the K131 beans

Sample	Tannin (mg/100 g)	Phytate (mg/100 g)
Control (raw)	931.79^a^ ± 0.29	137.59^a^ ± 3.54
DS	N/D	N/D
DR	N/D	N/D
WS	642.04^d^ ± 10.53	74.16^d^ ± 2.12
WR	892.20^b^ ± 12.08	112.24^c^ ± 2.83
Reference (boiled)	864.33^c^ ± 10.61	122.72^b^ ± 3.54

Means values ± SD of triplicate determinations, dry matter basis. N/D means not detected. Means within the same column with different superscripts are significantly different at *P* < 0.05.

DS, soaked-sprouted-Dehulled-Steamed; DR, soaked-sprouted-Dehulled-Roasted; WS, soaked-sprouted-Whole-Steamed; WR, soaked-sprouted-Whole-Roasted.

#### Protein digestibility

The protein digestibility of the bean samples ranged from 41.6% to 64.1% (Table2) and was higher for dehulled than whole beans.

**Table 2:** Effect of combined processing methods on in vitro protein digestibility of K131 beans

Sample	Crude protein (g/100 g)	% Protein digestibility
DS	29.94^a^ ± 0.14	64.08^a^ ± 0.19
DR	30.06^a^ ± 0.13	56.95^b^ ± 2.15
WS	28.52^b^ ± 0.10	51.75^b,c^ ± 3.03
WR	27.94^c^ ± 0.09	41.56^d^ ± 1.93
Reference (boiled)	27.63^c^ ± 0.27	49.61^c^ ± 2.72

Means values ± SD of triplicate determinations, dry matter basis. Means within the same column with different superscripts are significantly different at *P* < 0.05.

DS, soaked-sprouted-Dehulled-Steamed; DR, soaked-sprouted-Dehulled-Roasted; WS, soaked-sprouted-Whole-Steamed; WR, soaked-sprouted-Whole-Roasted.

#### Mineral extractability

Iron and zinc extractability were higher in dehulled beans than whole beans. Roasting resulted in greater increase in both iron and Zinc extractability. Beans that were dehulled-sprouted-roasted had the highest iron and zinc extractabilities (70.02% and 70.12%, respectively) (Table3). Iron extractability was least in boiled beans while zinc extractability was least in boiled and whole-sprouted-roasted beans.

**Table 3:** Effect of combined processing methods on iron and zinc extractability of the K131 beans

Bean sample	Iron (mg/100 g)	Iron extractability (%)	Zinc (mg/100 g)	Zinc extractability (%)
DS	14.37^bc^ ± 0.89	62.57^b^ ± 1.54	3.69^c^ ± 0.12	58.66^b^ ± 1.45
DR	13.04^c^ ± 1.01	70.02^a^ ± 0.58	3.86^c^ ± 0. 08	70.12^a^ ± 1.32
WS	16.73^ab^ ± 1.47	28.32^d^ ± 2.30	4.84^b^ ± 0.19	52.62^c^ ± 2.43
WR	15.47^bc^ ± 0.25	33.32^c^ ± 0.81	5.05^b^ ± 0.23	41.56^d^ ± 1.26
Reference (boiled)	19.46^a^ ± 1.55	5.76^e^ ± 0.42	5.67^a^ ± 0.08	42.61^d^ ± 0.93

Means values ± SD of triplicate determinations, dry matter basis. Means within the same column with different superscripts are significantly different at *P* < 0.05.

DS, soaked-sprouted-Dehulled-Steamed; DR, soaked-sprouted-Dehulled-Roasted; WS, soaked-sprouted-Whole-Steamed; WR, soaked-sprouted-Whole-Roasted.

A strong inverse relationship was observed between iron extractability and phytate levels (*r* = −0.932) as well as with tannin content (*r* = −0.911). Similarly, phytate and tannin contents had strong inverse relationships with zinc extractability (*r* = −0.927 and *r* = −0.921).

#### Total available carbohydrate

Samples that received moist heat treatment (steaming and boiling) had higher total available carbohydrates than those that were roasted (Table4).

**Table 4:** Effect of processing methods on the total available carbohydrate

Bean sample	Total available carbohydrate (%)
DS	43.09^ab^ ± 0.44
DR	39.14^bc^ ± 0.75
WS	48.56^a^ ± 3.80
WR	34.08^c^ ± 2.37
Reference (boiled)	46.28^a^ ± 2.62

Means values ± SD of triplicate determinations, dry matter basis. Means within the same column with different superscripts are significantly different at *P* < 0.05.

DS, soaked-sprouted-Dehulled-Steamed; DR, soaked-sprouted-Dehulled-Roasted; WS, soaked-sprouted-Whole-Steamed; WR, soaked-sprouted-Whole-Roasted.

Flours from beans that were roasted, that is, either sprouted-dehulled-roasted or whole-sprouted-roasted, had the lowest total available carbohydrate.

## Discussion

### Protocol for development of quick cooking bean flour

#### Soaking

The beans were soaked to facilitate water imbibition and aid sprouting. The weight of the soaked beans increased with time as water was imbibed during soaking. After 24 h minimal weight increase was observed as the beans reached their maximum water absorption capacity.

#### Sprouting

The selected sprouting time for all the beans was 48 h beyond which 70% of the sprouted beans began to germinate (developed well defined root hairs and a pale green color began to form at the point between the cotyledon and the radical). By that time, food reservoirs were likely being used for germination.

#### Steaming

The selected steaming time was when the beans were soft enough to be crushed between thumb and index figure. Dehulled beans took a shorter time to cook than whole beans due to absence of seed coats and probably faster water imbibitions and heat transfer.

#### Roasting

The roasted beans were golden brown in color had an average moisture content of 9.62% which is within the recommended range (below 12%) for production of flour. Whole beans required a longer roasting duration than the dehulled due to the presence of seed coats.

Significant elimination of tannins was achieved by dehulling implying that tannins were mainly in the seed coats. Dehulling was previously reported to substantially reduce the levels of tannins in beans (Alonso et al. 2000; Egounlety and Aworh 2003 and Ghavidel and Prakash 2007). However, beans that were soaked and sprouted followed by either steaming or roasting without de-hulling also had significantly lower tannin levels than those which were boiled whole. The difference could be attributed to leaching of tannins during soaking (Sangronis and Machado 2007) and the effect of heat (Barroga et al. 1985; Alonso et al. 1998) as well as polyphenolase enzyme's hydrolytic activity on tannins during sprouting (Reddy et al. 1985). Tannin levels were lower in flours from beans that were soaked, sprouted and steamed without de-hulling compared to flours from beans that were soaked, sprouted, and roasted without dehulling, implying that steaming caused a greater reduction in tannins compared to roasting. However, both steaming and roasting were more effective for reducing tannins than boiling. Cooking was reported to reduce tannins in black grams, red kidney, and white kidney beans (Rehman and Shah 2005; Wang et al. 2010) as well as in mung bean seeds (*Phaseolus aureus* L.) (Mubarak 2005), possibly through polymerization (Van der Poel et al. 1991) and changes chemical reactivity of the tannins (Barroga et al. 1985).

#### Phytate content

All bean samples (except the raw and boiled) were soaked to facilitate sprouting. However, in addition, soaking was previously reported to reduce Phytates (Beleia et al. 1993; Jood et al. 1997; El Maki et al. 2007) due to leaching of phytate ions into the soaking water. Water imbibition also activates phytase enzymes present in the beans, to degrade and reduce phytates (Vijayakumari et al. 2007). The increase in phytase activities continues during sprouting or germination (Bau et al. 1997), leading to further hydrolysis of phytic acid to inorganic phosphate and inositol (Eskin and Wiebe 1983). In this work, the elimination in phytates to below detection levels after dehulling implies that the phytates are concentrated in the hulls.

Steaming resulted in a greater reduction in phytates than roasting. Flour from whole beans that were sprouted and steamed had lower phytate levels than those that were similarly treated but roasted instead of steaming. The method of heat treatment was previously reported to affect the extent of phytate reduction (Udensi et al. 2007). The whole beans that were boiled had the highest phytate levels among the cooked samples. This implies that boiling whole, which is the commonest way of preparing beans, achieves limited reduction in phytates. However, whole raw beans had higher phytate levels than those that were boiled whole, implying that boiling also achieves some reduction in phytates.

Osman (2007) similarly reported that sprouting beans for 48 h reduced phytates by 48.94%, while roasting and cooking presoaked beans reduced phytates by 60.69% and 44.85%, respectively.

#### Protein digestibility

Higher protein digestibility in dehulled samples was probably because antinutrient levels (tannins and phytates) were reduced to below detection, reducing their negative influence on protein digestibility. Interaction of tannins and phytates with protein increases the degree of protein cross-linking, decreasing the solubility of proteins and making protein complexes that are less susceptible to proteolytic attack (Cheryan 1980; Reddy et al. 1985). Protein digestibility is also affected by the fiber content (Hughes et al. 1996) and reduced fiber content may have contributed to the higher digestibility of dehulled beans.

Roasting reduced protein digestibility of beans that were dehulled, sprouted and roasted compared to those which were similarly treated but steamed instead. This could be due to interaction with tannins and phytates (which were higher in roasted samples) (Cheryan 1980; Reddy et al. 1985). Moist heat (such as boiling and steaming) results in products with higher in vitro protein digestibility (IVPD) than dry heat treatment (such as roasting) (Giami et al. 2001; Osman 2007). There was a strong negative correlation (*r* = −0.854 and *r* = −0.87, respectively) between protein digestibility and phytates and tannins content.

#### Mineral extractability

Higher mineral extractability in dehulled samples was probably because phytate and tannin levels were reduced to below detection. Boiled beans had the lowest iron extractability possibly because of higher phytate levels. As a divalent cation, iron, is generally associates with phytic acid possibly reducing its extractability (El Maki et al. 2007). Soaking reduces phytic acid, freeing iron, and resulting in higher HCl extractability (Duhan et al. 2002; Ghavidel and Prakash 2007).

Combined processing (sprouting, dehulling followed by either roasting or steaming) of beans resulted in higher iron extractability than it did for Zinc. Contrary to Duhan et al. (2004) who reported greater zinc extractability of beans that were soaked, germinated and dehulled compared to those that were ordinarily cooked, there was no significant difference between Zinc extractability of beans that were soaked-sprouted-whole-roasted and those that were boiled whole. This may be due to the higher tannin content in whole-sprouted-roasted beans flour compared to the rest of the processed bean flours.

A strong inverse relationship between iron extractability and phytate levels as well as with tannin content implies that extractability of iron increases with removal of phytates and tannins from the beans. Tannins easily chelate iron, reducing its extractability (Lestienne et al. 2005). Processes that reduced tannins, (soaking, dehulling, and sprouting) therefore also improved iron extractability. Similarly, strong inverse correlations of phytate and tannin contents with zinc extractability implied that reduction in phytate and tannin contents led to significant increase in zinc extractability of the bean flours.

#### Total available carbohydrates

Lower total available carbohydrates in roasted beans may be due to the conversion of carbohydrates into degradation products during roasting (Oosterveld et al. 2003). Frias et al. (1999) reported that dry cooking caused higher reduction in total available carbohydrates in chickpea as compared to cooking (boiling).

## Conclusion

A protocol for processing bean flour was developed. A combination of soaking, sprouting, and dehulling followed by heating generally increased nutrient digestibility and bioavailability. Dehulling effectively removed tannins and phytates, increased iron and zinc availability and increased protein digestibility. It is therefore a key step to include in the combined processing of beans for the nutritionally vulnerable. Consumption of the processed beans developed in this work has the potential to increase nutrient intake and improve the nutrition and status of the vulnerable. The processed bean flours require less cooking time and less fuel than whole beans. They therefore have the potential to reduce drudgery and contribute to environment conservation.
